# Patient Flow Analysis in Resource-Limited Settings: A Practical Tutorial and Case Study

**DOI:** 10.9745/GHSP-D-14-00121

**Published:** 2015-03-02

**Authors:** Cinnamon A Dixon, Damien Punguyire, Melinda Mahabee-Gittens, Mona Ho, Christopher J Lindsell

**Affiliations:** a University of Cincinnati, Cincinnati Children's Hospital Medical Center, Division of Emergency Medicine, Cincinnati, OH, USA; b University of Cincinnati, Cincinnati Children's Hospital Medical Center, Center for Global Health, Department of Pediatrics, Cincinnati, OH, USA; c Kintampo Municipal Hospital, Kintampo, Ghana; d University of Cincinnati, College of Medicine, Department of Emergency Medicine, Cincinnati, OH, USA

## Abstract

Patient flow analysis (PFA), a simple quality improvement tool to identify patient flow patterns, can be used in resource-limited settings to inform service delivery improvements. A PFA at a Ghanaian hospital found that personnel constraints and a mismatch between staffing and patient arrival surges led to long wait and total attendance times. The median time from arrival to first-provider contact was 4.6 hours.

## INTRODUCTION

Overcrowding of health care facilities and inefficient patient flow affect quality and timeliness of care,^1^ as well as patient satisfaction.^2^^,^^3^ The mismatch of supply and demand, often experienced in high-volume care settings, such as emergency departments (EDs) in developed countries, is amplified in medical facilities of low- and middle-income countries (LMICs), where they are burdened by a high volume of patients while coping with limited resources and staffing.

Overcrowding and inefficient patient flow at health care facilities affect the quality and timeliness of care, as well as patient satisfaction.

Patient Flow Analysis (PFA) is a quality improvement tool which can be used to help identify patient flow inefficiencies at any type of health care facility and inform areas for intervention to help improve care delivery processes.^4^^–^^8^ This low-technology methodology involves 2 important aspects: *flow mapping*—which provides a qualitative perspective of the process of care delivery, and *cycle-time measurement*—which provides quantitative data on time throughout the patient's entire care delivery process.^9^ Both aspects of PFA help investigators identify key constraints in flow as well as gaps in staffing and resources in the unique health care setting. Additionally, when arrival and throughput data are mapped to resource allocation, such as staffing availability, mismatches can be identified and can further inform reallocation or redirection of resources, or application of new resources. In the ideal setting, PFA is a part of a larger health care improvement initiative, and the findings lead to subsequent identification, implementation, and testing of interventions for improved care delivery.

Patient Flow Analysis uses qualitative and quantitative data to identify areas for improvement in patient care delivery.

Although PFA is regularly used in developed countries to help evaluate wait times and improve efficiency and patient care, it has been applied minimally in health care facilities of LMICs despite the opportunity for improving care systems in those environments. This article provides a basic tutorial on how to perform a PFA in a resource-limited setting and highlights a case study with lessons learned from a PFA performed in one Ghanaian municipal hospital.

## CONDUCTING A PATIENT FLOW ANALYSIS

Every PFA is unique, and variation exists in the process. Regardless of the study setting, however, a step-wise approach can be helpful in implementing a successful PFA. The following suggested steps, which we have termed the 3Ps—preparation, piloting, and performing—can be used as a flexible guide of items to consider when performing a PFA. Importantly, after performing a PFA, the collected data should be analyzed as it relates to the objectives and process measures to help identify areas for improvement.

### Phase 1: Preparation

While preparation may be informal, this aspect is an important first step to implementing a PFA. This period often entails processes such as building a team, understanding the system in which the PFA will occur, defining the objectives and process measures of the study, determining the study plan, developing PFA documents and ensuring that necessary equipment is available, identifying and training key personnel, and briefing the staff. Because none of these steps are mutually exclusive, it is helpful to have at least one investigator involved in all aspects.

- **Building a team:** Although one individual can define study objectives and process measures as well as identify potential challenges prior to PFA implementation, it is often advantageous to have 2 or more stakeholders contribute to this process. Ideally, this team will include leaders within the clinical environment and will represent different skill levels and work areas, helping foster support from the staff and thus influencing the success of the PFA.
- **Understanding the system:** Understanding the current system prior to commencing the PFA is important. Flow mapping or process mapping prior to performing the PFA helps depict potential pathways of current patient care. Additionally, it can highlight activities at each stage in a patient journey and identify current needs in terms of staffing and other resources. This flow mapping can be either a hand-drawn or computerized diagram.
- **Defining objectives and process measures:** Defining objectives to be addressed and process measures to be identified helps guide PFA methodology and the duration of the study. Although objectives and measures vary depending on the specific setting, most are time-based, such as determining the length of stay and/or length of queuing, or are capacity-based, such as patient arrival per hour and/or patient-staffing mismatch. Within these, it is also important to consider which patients the PFA will track and the times at which the PFA will occur.
- **Determining study plan:** Three time-based elements exist within most PFAs: (1) patient arrival time into the system (patient enters the clinical setting), (2) times between and at each stage of the patient care process, and (3) patient discharge time out of the system. Documentation of this time-based data occurs throughout the patient journey. Thus, most PFAs have patients carry a form throughout their visit, which is collected when they exit the system. Additionally, many PFAs have an observational aspect of the study in which investigators or personnel document qualitative observations regarding flow and processes. Determining the duration of the PFA can be challenging. The ideal duration is long enough to account for variations in patient surges/acuity, times/days, and staffing number/skill sets, and yet it remains short enough so that staff and key personnel are not overburdened and the PFA itself does not become part of the regular system.
  PFAs should be long enough to account for variations in patient surges, times/days, and staffing, yet short enough to avoid overburdening personnel and making the PFA part of the regular system.
- **Development of study documents and ensuring available equipment:** Study documents should fit the clinical environment and defined process measures. Time logs can be developed or modified to assist in documentation. Equipment needed for a PFA is minimal: a method for synchronized time documentation throughout the clinical environment (e.g., synchronized timers or watches stationed at each location), pencils/pens for documentation, and possibly a stapler to attach forms.
- **Identifying and training key personnel:** While it is possible for one individual to perform a PFA, having 2–3 key personnel who can assist during the PFA is extremely helpful. These individuals can help with timer synchronization, arrival documentation, collection of forms, and other activities. Training for these individuals is minimal but necessary to ensure that they follow the same system.
- **Briefing the staff:** Since PFAs happen within a functioning clinical environment, it is valuable to brief the staff about the study. In many cases, hospital staff are part of the PFA process, for example, by filling out time logs. While simple, this data collection aspect can add additional time and work for these individuals, thus having their buy-in is important. It helps to establish a defined time for staff to learn about the project, train in data collection if they are to assist, and ask questions and provide feedback. While the “Hawthorne effect” of improved or modified performance of staff while being observed is possible, these briefings should encourage staff to go about their normal daily work processes, in order to get an accurate perspective of the system.

### Phase 2: Piloting

Although piloting the PFA is not mandatory, it can be very helpful in revealing potential issues or flaws within the study plan and/or areas for improvement. Furthermore, piloting the PFA allows investigators to review pilot data to determine its accuracy and decide whether a retraining session is necessary.

### Phase 3: Performing

Performing the PFA is simple if it has been well planned and prepared, and piloted. The PFA should commence each day with a sufficient number of documents to cover the anticipated number of patients, and timers/watches should be verified to function and should be synchronized. Unforeseen circumstances can and do occur; however, these variances are important to understand and document. Key personnel should document observational findings throughout the PFA, with notations of time/date and descriptors of the event.

Two important aspects to consider during the PFA are the well-being of staff and the patients. Key individuals should have regular, scheduled breaks; however, ideally there should be no lapse in time or coverage of the individual's task (for example, if a key individual is documenting arrival times, there should be a transfer of that task to another individual prior to the break). Lastly, patient safety should always come before the PFA. If any personnel or staff feels the need to circumvent the PFA for the sake of patient safety/care, this should be done and observational documentation can follow.

Personnel should know that they can circumvent a PFA for the sake of patient safety.

## A CASE STUDY FROM GHANA

We conducted a PFA at Kintampo Municipal Hospital, a district-level hospital in central Ghana that serves a population of over 100,000 individuals. The care model in this medical facility is similar to that of the acute care model of most US-based EDs: there are no scheduled appointment times, and evaluations typically occur on a first-come, first-served basis. Unlike US EDs, however, which operate 24 hours per day/7 days a week, the hours of operation for acute care in this facility are typically weekdays, between 5 am–5 pm. During these times, the general process of care involves registration, history taking, provider care, ancillary testing, and disposition.

This PFA quality improvement study protocol was reviewed by the institutional review board at Cincinnati Children's Hospital Medical Center and deemed not human subjects research.

### Phase 1: Preparation for the Ghanaian Municipal Hospital PFA

The objectives of this PFA were to identify key constraints to patient flow, discordances between demand for service and staffing, and opportunities for increasing efficiency. Process measures included total visit time, time at and between each stage of care, patient arrival patterns, and staffing patterns. Our overarching goal was to use findings from this PFA to identify potential interventions that could improve care delivery at this hospital. We were unable to perform the anticipated follow-up PFA to evaluate such interventions due to unforeseen resource constraints.

**FIGURE 1.**
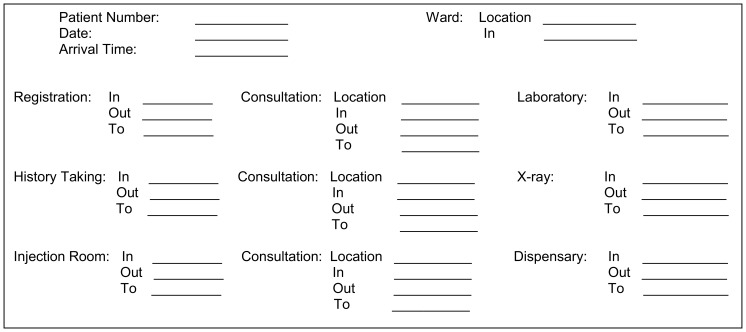
Patient Flow Analysis Time Log

To facilitate collection of time-based data throughout the patient's hospital visit, we developed a modified time log from previously published time logs^4^^,^^5^^,^^7^ and purchased 15 basic watches (<US$1 each) at the local market. Time logs were used to document each patient's initial arrival time to the facility, time in/out of every stage within the hospital course, and final disposition (Figure 1). Watch times were synchronized, and one watch was stationed and secured at each hospital location.

Hospital staff and key personnel were briefed and trained on the project one week prior to the PFA. During this 30-minute educational session, hospital staff learned the objectives and methodology of the project and how to use the time log. Lunch was provided.

### Phase 2: Piloting the Ghanaian Municipal Hospital PFA

We implemented a 1-day pilot test of data collection several days prior to the PFA, with review of data for discrepancies. Follow-up retraining of personnel occurred immediately following the pilot data collection period.

### Phase 3: Performing the Ghanaian Municipal Hospital PFA

Data collection occurred from June 29–July 3, 2009, with real-time patient flow data collected for all patients arriving during typical hospital hours. Upon presentation to the hospital and prior to registration, each patient received a time log on which the arrival date and time were documented. At registration, the time logs were attached to patients' medical charts where they remained throughout their hospital visit. Patients presented their medical charts with attached time logs to hospital personnel at each hospital location. Time logs were purposely printed on bright green paper to increase their visibility on the patient's medical chart. Using study watches (synchronization confirmed twice daily), hospital staff documented the times in and out of each hospital location, as well as the next anticipated location. Time logs were collected at the end of the patient's visit.[Fig f03]

Hospital personnel recorded patients' progress in and out of each location using synchronized watches and time logs.

**Figure f03:**
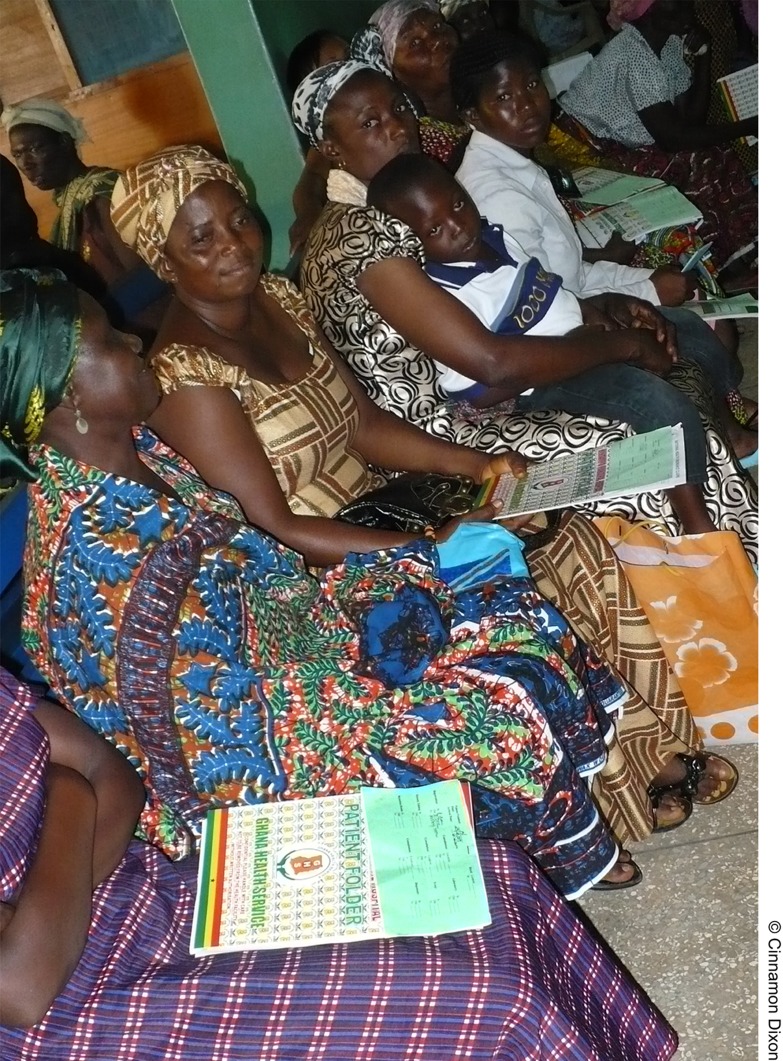
At Kintampo Municipal Hospital in Ghana, patients wait for medical care, with green patient flow analysis time logs attached to their medical charts. Time logs were purposely printed on bright green paper and stapled to the front of patients' charts to increase their visibility.

We quantified hospital staffing and infrastructure by direct observation throughout the study. The number of hospital personnel and the hours of operation of 3 critical patient contact areas—registration, history taking, and consultation rooms—were recorded. Investigators also documented other process-related items that could affect patient throughput.

### Data Analysis

PFA data were analyzed using SAS v. 9.3. Median and mean duration times at each stage, between stages, and total duration were calculated. The use of median is important for reporting central tendency in skewed time flow data. Complementing this with use of the mean emphasizes the outliers, which have a significant effect on patient flow, care quality, and satisfaction. Student's t-test was used to test for differences between patients that were admitted or discharged home.

## RESULTS

Of the 564 time logs given to patients presenting to the hospital during this study, 487 (86%) were collected. Complete data, describing the patient's flow through their hospital visit, were available in 74% (362) of time logs, and partial data for 17% (81) of time logs. We excluded all data from potentially flawed time logs (9%, or 44 participants), which were defined as those having negative time points and those with data occurring over multiple days or visits.

The mean number of patients arriving to the hospital per hour during the study was 10.5 (standard deviation [SD] = 7.1). Peak arrival occurred during the morning hours—between 7 am and 9 am (Table 1). The large majority (96%) of patients were discharged home after completion of their hospital visit; 4% were admitted.

**TABLE 1.** Number of Patients Arriving to the Hospital per Hour

**Arrival Hour**	**Mean**	**SD**	**Median**	**Minimum**	**Maximum**
05:00–05:59	7.2	1.5	7.0	5.0	9.0
06:00–06:59	12.4	2.3	12.0	9.0	15.0
07:00–07:59	18.6	5.7	18.0	13.0	27.0
08:00–08:59	18.6	3.5	19.0	15.0	22.0
09:00–09:59	15.2	7.2	16.0	5.0	25.0
10:00–10:59	12.4	6.9	11.0	6.0	24.0
11:00–11:59	4.6	3.0	4.0	1.0	9.0
12:00–12:59	4.4	2.3	4.0	2.0	8.0
13:00–13:59	1.7	0.6	2.0	1.0	2.0
14:00–14:59	4.0	0.0	4.0	4.0	4.0
15:00–15:59	3.0	1.4	3.0	2.0	4.0

Abbreviation: SD, standard deviation.

**FIGURE 2.**
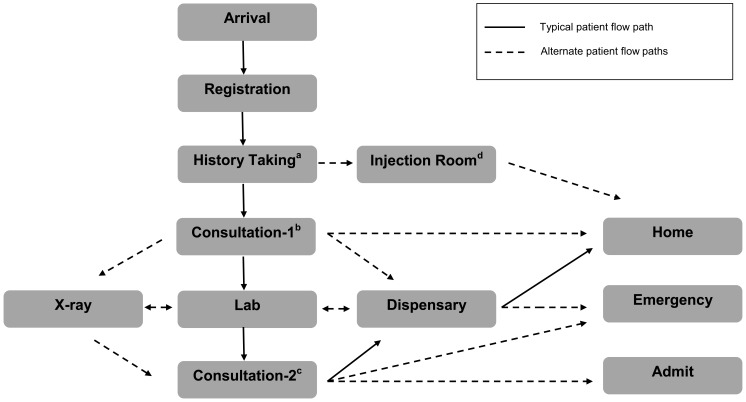
Possible Patient Care Flow Paths Through the Kintampo Municipal Hospital in Ghana ^a^ History Taking: Nurse solicits chief complaint and obtains patient's temperature and blood pressure. ^b^ Consultation-1: First consultation with a medical provider (physician or medical assistant); history and physical performed, ancillary tests ordered, and/or the patient is discharged home via the dispensary. ^c^ Consultation-2: Second consultation with a medical provider (depending on the time of day and availability of medical providers); if there is no second consultation, the patient is sent directly from ancillary testing to the dispensary for medications and discharged home. ^d^ Injection Room: Patients undergo scheduled treatment for chronic diseases (e.g., tuberculosis).

Patients experienced several different care paths throughout their hospital visit. Figure 2 highlights the most common care path. The overall total median visit time from arrival to disposition was 5.2 hours (interquartile range [IQR] = 4.1–6.2 hours; mean = 5.1 hours, SD = 1.6). This duration did not differ significantly between admitted and discharged patients (*P* = .11). Additionally, median time between arrival and first-provider contact was 4.6 hr (IQR = 3.4–5.6 hours; mean = 4.4 hours, SD = 1.6). Longest wait times were between arrival and registration (median = 2.3 hours, IQR = 1.4–3.2 hours; mean = 2.2 hours, SD = 1.3) and between history taking and first-provider contact (median = 1.4 hours, IQR = 0.7–2.1; mean = 1.5 hours, SD = 1.1). Other long delays were noted between registration and triage, and between laboratory testing and processing (Table 2).

**TABLE 2.** Duration In and Between Hospital Locations (in Hours)

**In/Between Location**	**Mean**	**SD**	**Median**	**IQR**	**Minimum**	**Maximum**
Registration	0.15	0.19	0.12	0.08–0.18	0	3.10
History taking	0.07	0.07	0.07	0.05–0.08	0	1.03
Consultation-1	0.12	0.19	0.08	0.07–0.12	0	2.13
Consultation-2	0.07	0.10	0.05	0.03–0.08	0.02	0.83
Laboratory	0.79	0.56	0.72	0.45–0.97	0.05	2.82
X-ray	0.24	0.11	0.24	0.17–0.32	0.17	0.32
Dispensary	0.14	0.87	0.07	0.03–0.12	0	17.15
Arrival – Registration	2.24	1.25	2.30	1.35–3.22	0.02	6.47
Registration – History taking	0.65	0.55	0.45	0.23–0.92	0	2.55
History taking – Consultation-1	1.51	1.10	1.42	0.73–2.14	0	12.07
Consultation-1 – Laboratory	0.13	0.13	0.08	0.03–0.15	0.02	0.78
Laboratory – Consultation-2	1.00	2.70	0.29	0.12–0.53	0.02	15.17
Consultation-2 – Dispensary	0.17	0.14	0.13	0.08–0.24	0	0.62
Laboratory – Dispensary	0.52	0.67	0.28	0.12–0.59	0.08	2.08
Consultation-1 – Dispensary	0.22	0.30	0.15	0.07–0.28	0	3.15
TOTAL	5.06	1.58	5.18	4.07–6.20	0.60	9.42

Abbreviations: IQR, interquartile range; SD, standard deviation.

Table 3 describes hospital staffing over the study period. In general, although patient arrival peaked in the early morning hours, maximum staffing for the 3 patient contact areas assessed (registration, history taking, and consultation rooms) occurred only after this surge. This discrepancy had a cumulative effect for all other patients, as delays accumulated throughout the day. Additionally, the medical consultation rooms were open for only brief periods each day (mean = 3–5 hours), and closed to patient care at similar times of day, resulting in many patients not being seen at all. Consultation rooms often closed regardless of patient volume or the presence of waiting patients (Box).

**TABLE 3.** Observational Findings of Hospital Staffing and Hours of Operation at Kintampo Municipal Hospital

**Hospital Location**	**Range of Opening Times**	**Staff Number**	**Staffing Hours**	**Range of Closing Times**	**Total Daily Patient Care Hours (mean)**
Registration	05:45	1	05:30–08:00	14:00–16:00	9
		2	08:00–09:00		
		3	After 09:00		
History Taking	08:00	1	08:30–09:00	14:45	7
		2	After 09:00		
Consultation Room 1	09:30–11:00	1		14:00–16:00	4.2
Consultation Room 2	10:00–11:30	1		13:00–15:00	3.3
Consultation Room 3	08:15–10:00	1		12:45–15:00	5.4
Consultation Room 4	11:00–12:00	1		14:00	3.0^a^
Laboratory	07:00				
Dispensary	08:30				

a Medical provider for Consultation Room 4 was not present on 3 days of the patient flow analysis; data available for only 2 days.

**BOX. Process-Related Observational Findings From the Kintampo Municipal Hospital Patient Flow Analysis**

- Unclear arrival procedures.
  - Many patients uncertain as to where registration line starts/ends.
  - Information provided to patients by non-hospital personnel (e.g., other patients), often resulting in patients receiving misinformation (e.g., patients waiting in wrong lines).
- Patient flow exceeds seating capacity; patients sit on floors while waiting.
- No process for initial triage or recognition of severely ill patients.
- Patients that are approached about or enrolled in research studies have different flow processes or are moved ahead in the flow process.
- Storage of supplies affects delivery of care (e.g., hospital staff unable to open history taking because head nurse has key for where blood pressure cuff is stored).
- Patient volume outstrips hospital staffing and hours.
  - Registration closes based on physician determination, not patient volume.
  - Consultation rooms close regardless of patient volume or waiting patients.
- Communication lapses.
  - Staff performing history taking not aware of consultation room closures (brief or extended).
  - Patients directed to closed consultation rooms.

## DISCUSSION

Health care facilities worldwide face the challenge of providing high-quality care while struggling with large patient volumes and process inefficiencies. PFA is a quality improvement tool that can help identify critical constraints in patient flow at any type of health care facility and improve allocation of resources. A step-wise approach considering items within the 3Ps—preparation, piloting, and performing—can be helpful in implementing a successful PFA.

Our case study demonstrates the utility of the PFA tool in the resource-limited setting of Ghana. We found that patients experienced lengthy hospital visits, with a median duration from arrival to disposition of 5.2 hours (independent of disposition). While comparison data from other LMICs are not publicly available, the total hospital visit duration in our case was significantly more than the US ED median duration of 3.2 hours.^10^ Additionally, there were extensive delays prior to medical evaluation; most notably, between arrival and first-provider contact (median = 4.6 hours). This “door-to-provider” time, which is an important quality indicator in other acute care settings, is nearly 6 times the US median time of 46 minutes.^10^ Although the implication of long durations of patient care in this setting has not been evaluated, we would expect effects similar to other acute care models where longer lengths of stay and total hospital durations are associated with increased morbidity, delays in definitive care, and poorer overall perceptions of care.^11^ Furthermore, we would anticipate that significant delays prior to medical evaluation would be even more detrimental in an environment where acuity level is not assessed upon presentation to the facility and no formal triage exists.

Patients at the Ghanaian hospital experienced lengthy hospital visits and door-to-provider delays that were nearly 6 times the US median time of 46 minutes.

Results from our direct observational assessment showed important inefficiencies and process-related items. Most notably, there was considerable mismatch between demand for and supply of services, which was amplified by daily inconsistencies in staffing and hours of operation and by miscommunication about staff availability.

There was considerable mismatch between demand for and supply of services.

Several subsequent changes in the care process at the Ghanaian Municipal Hospital have occurred since the PFA, including designated registration areas for differing patient populations, decreased duplications of history taking within the system, an increase in the number of consultation rooms, and the addition of some staff in selected areas.

### Lessons Learned

Three main lessons were learned from implementing this PFA:

- **Increasing the study duration helps account for variation and potential lost patients**. Our study period was limited to one midsummer time period. Although 87% of charts were collected at the end of the study, it is unknown whether those patients who left the facility without care were also those most likely to not have forms collected at the end of their visit, and therefore were underrepresented in the study.
- **Having a more systematic observational assessment of hospital staffing and infrastructure can improve the strength of resource allocation recommendations.** While our study relied upon descriptive data, which is consistent with anecdotal descriptions of the staffing culture at this facility, a more robust evaluation of staffing would have strengthened our findings and recommendations.
- **Implementing changes based on recommendations with a follow-up PFA is critical to determining improvement.** Our case study is substantially limited by not having evaluated specific interventions after performance of the PFA. Despite this, the results have led to improved knowledge and culture competency around the importance of efficient patient flow, and several changes to the patient care process have been implemented subsequent to the PFA.

## CONCLUSION

PFA is a simple, low-resource tool useful in evaluating health delivery processes and identifying areas for intervention. When ideally used within a larger health care improvement initiative, in combination with intervention implementation and follow-up evaluation, PFA can improve quality of care in resource-limited settings.

## Notes

**Peer Reviewed**
